# Tracheobronchial amyloidosis in primary Sjögren syndrome: A case report

**DOI:** 10.1097/MD.0000000000022942

**Published:** 2020-10-23

**Authors:** Jiangyun Luo, Yongpeng Ge

**Affiliations:** a Department of Rheumatology and immunology, the second people's Hospital of Ningxia; b Department of Rheumatology, China-Japan Friendship Hospital, China.

**Keywords:** Sjögren syndrome, amyloidosis, Congo red

## Abstract

**Rationale:**

Tracheobronchial amyloidosis (TBA) associated with Sjögren syndrome is very rare. Here, we describe a case with this phenomenon, in order to better understand the condition.

**Patient concerns:**

A 52-year-old woman presented after 6 months of coughing, sputum, and dyspnea. Chest computed tomography revealed thickened bronchial walls, which were irregular on the left side the trachea. She had a history of dry eye and dry mouth of at least 3 years’ duration.

**Diagnoses:**

Sjögren syndrome was diagnosed based on her symptoms, ophthalmological and parotid examination, and immunological and autoantibody tests. The diagnosis of TBA was confirmed by Congo red staining of a tracheal biopsy.

**Interventions:**

The patient was given glucocorticoids without any other immunosuppressants.

**Outcomes:**

The symptoms improved after 6 months.

**Lessons:**

TBA associated with Sjögren syndrome is a rare condition. TBA is characterized by amyloid deposition to the trachea in the absence of systemic amyloidosis. Diagnosis requires tissue biopsy with demonstration of amyloid deposition.

## 1 Introduction

Sjögren syndrome is an autoimmune disease characterized by chronic inflammation of the systemic exocrine glands and symptoms of dryness owing to their impaired function. Extra-glandular organs such as the trachea and the lungs may also be involved.^[1]^ Amyloidosis is a systemic disease caused by the accumulation of amyloid deposits in the whole body. Tracheobronchial amyloidosis (TBA) is extremely rare with very few reports of its occurrence together with Sjögren syndrome.^[2–3]^ Here, we describe such an extremely rare case of TBA associated with Sjögren syndrome.

## 2 Case presentation

Written informed consent was obtained from the patient for publication of this case report and accompanying images. The study was approved by the Ethics Institutional Review Board of the China-Japan Friendship Hospital. A 52-year-old woman was admitted to our institution with a cough, sputum, and dyspnea on exertion which had lasted for 6 months. History of present illness: Six months earlier, the patient had developed dyspnea, cough and sputum accompanied by fever, with a maximum temperature of 38.5°C and no hemoptysis. One month later, she was admitted to a local hospital and given anti-infection treatment for 3 weeks. Her temperature returned to normal. Symptoms of cough, sputum and her difficulty in breathing eased slightly. Three weeks before her referral to our hospital, the dyspnea worsened again, and she was sent to us for further diagnosis and treatment. The patient had suffered from dry eyes and mouth for at least 3 years, but without any skin rash, joint pain, hair loss or oral ulcers. The patient was not medically treated for these symptoms.

She reported no smoking, alcohol consumption, illicit drug use, or occupational exposures. She had a family history of cancer, having suffered cervical cancer 11 years previously which was surgically resected. There was no history of hypertension, diabetes, or coronary heart disease.

Vital signs included a blood pressure of 115/76 mm Hg, a heart rate of 78 beats/min, a respiratory rate of 20 breaths/min, and an oxygen saturation of 93% in indoor air. Cardiac examination was normal. Pulmonary examination revealed clear breathing, no rales, wheezes, or bronchi. Neurologic examination was unremarkable. Oral examination revealed dental caries.

Laboratory blood values indicated leukopenia (white blood cells 2.41 × 10∗9/L) and thrombocytopenia (platelets 69 × 10∗9/L), hypergammaglobulinemia (IgG 2720 mg/dL), hypocomplementemia (complement C_3_ 66 mg/dL, C_4_ 15.6 mg /dL), presence of autoantibodies such as anti-SSA, anti-Ro52 antibodies, an elevated antinuclear antibody titer of 1:100, and an elevated rheumatoid factor titer (1600 IU/mL). Infectious pulmonary workup was negative. Transthoracic echocardiography, metabolic panel, tumor markers, urinalysis, urine protein to creatinine ratio, and serum/urine protein electrophoresis were all unremarkable.

The results of Schirmer test and tear film breakup time were consistent with xerophthalmia. Parotid emission computed tomography revealed impaired bilateral salivary gland function. The patient had a score >10 foci/4 mm^2^ for focal lymphocytic sialadenitis.

**Figure 1:**
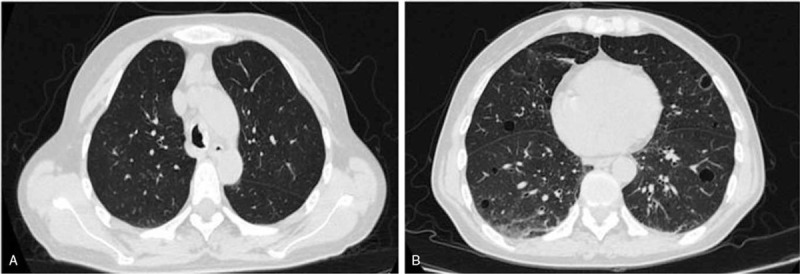
A. Chest CT scan revealed the mass in the lumen of trachea. B. Ground glass shadows, thin walled cavity and multiple nodule shadows.

**Figure 2:**
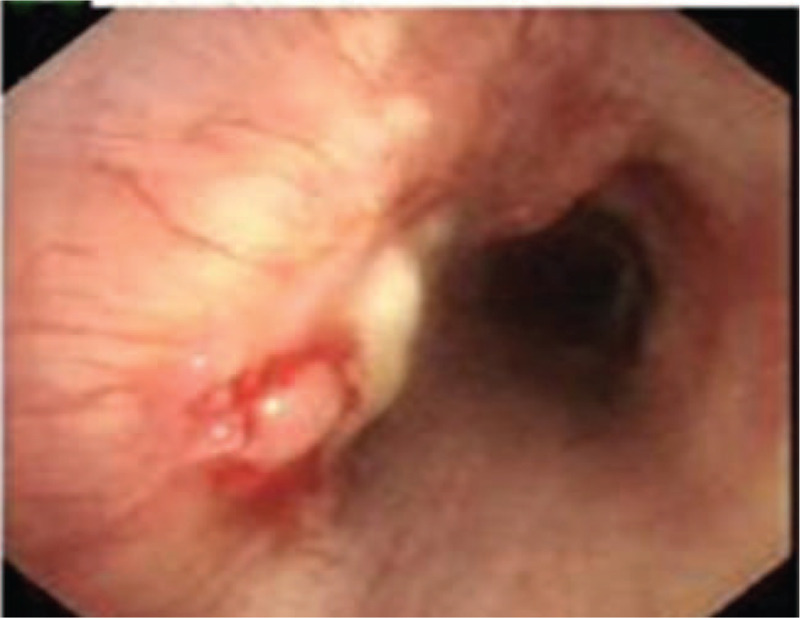
Endoscopic visualization shows the mass protruding from the superior left lateral wall of the carina.

Chest computed tomography revealed thickened bronchial walls, which were irregular on the left side the trachea (Fig. 1A). Scattered secular shadows, ground glass shadows, and thin-walled cavity, multiple nodule shadows of different sizes in the anterior mediastinal and thymus area, and multiple enlarged lymph nodes in the mediastinal and bilateral axillary were also apparent (Fig. 1B). Lung function suggested reduced diffuse function (forced expiratory volume in 1 second/forced vital capacity was 60.93%, and lung diffusing capacity for carbon monoxide was 56.2%). Bronchoscopy showed a mass on the left side of the lower end of the trachea, with a length of about 2.5 cm. The surface of the mass was uneven, with fluorescence showing crystal red, and the opening of the left main bronchus was partially covered (Fig. 2).

Results from the tracheal biopsy showed that large amounts of amyloid deposits with calcification and ossification were present in the interstitium, and Congo red staining was positive. The specimens were immunohistochemically positive for κ chains, but negative for λ chains. The patient was therefore diagnosed with TBA associated with Sjögren syndrome.

The patient was initially given intravenously methylprednisolone treatment at 40 mg daily, and then changed to oral methylprednisolone after two weeks, after which the dose was gradually tapered off. No other immunosuppressants were given in combination with the methylprednisolone. No serious adverse reactions such as infection were recorded. The patient's symptoms improved after 6 months. Her HRCT showed that the tracheal mass was smaller than before, the ground glass shadow disappeared, and the nodular shadow decreased. At the present time, the patient has been followed up for 12 months and remains in a stable condition.

## 3 Discussion

Amyloidosis is a disease of abnormal beta-pleated protein sheet deposition. It is classified into systemic and organ-specific types. Organ-specific amyloidosis is almost always caused by locally produced protein precursors.^[4]^ The respiratory system is rarely involved as an exclusive site of amyloid deposition, but amyloid plaques which also deposit in the airway submucosa can lead to TBA. TBA is much less common than lung amyloidosis. Our case was a 52-year-old woman, consistent with findings in the literature that amyloidosis commonly affects middle-aged people (50–60 years old).^[5]^ The common presenting symptoms of TBA include cough, wheezing, dyspnea, hemoptysis, and stridor.^[6]^ The patient reported here first suffered cough and sputum; after that, progressive dyspnea added to her complaints.

Amyloidosis in Sjögren syndrome patients is a rare association; it had been reported in only a few papers. Thus, Hanine Inaty et al reported a 57-year-old man with a history of Sjögren syndrome and a biopsy-proven TBA, who presented at the hospital with a 4 week history of fever, night sweats, cough, left-sided pleuritic chest pain, and unintentional weight loss.^[2]^ However, the frequency of TBA in patients with Sjögren syndrome is unknown. To characterize the patients with respiratory amyloidosis occurring in Sjögren syndrome, Takeshi Saraya et al reviewed the relevant literature on Japanese patients, with only three cases. Among all the patients that they evaluated, the proportion with TBA was 23.1%.^[3]^ Another review analyzed the literature regarding amyloidosis in association with Sjögren syndrome published up to April 2016. This analysis identified 55 cases, of which 25 had lung amyloidosis and 2 had TBA. The proportion of TBA in respiratory amyloidosis in Sjögren syndrome patients was thus only about 7.4%.^[7]^ In their review, they also described three patients with systemic involvement that included bone marrow biopsy with 7% to 15% of plasma cells. Other organs with localized amyloidosis were the skin (n = 19), kidney (n = 1), tongue (n = 1), breast (n = 2), salivary gland (n = 1), and vocal cord (n = 1). Most of the localized forms corresponded to the AL type. The diagnosis of amyloidosis followed the onset of the Sjögren syndrome by 1 to 25 years, and hypergammaglobulinemia, positive RF and/or anti-SSA and anti-SSB antibodies were observed in the majority of patients.^[7]^

The present case illustrates TBA as an extremely rare phenomenon in Sjögren syndrome. Endoscopically, TBA can present as submucosal plaques, nodules, polypoid lesions protruding into the lumen of the airway, or rarely circumferential wall thickening. There may be different endoscopic forms coexisting in the same patient. In the current case report, it is unclear whether TBA co-occurred with or was caused by Sjögren syndrome. However, the patient suffered TBA appearing 3 years after Sjögren syndrome onset, and both Sjögren syndrome and the TBA were improved after glucocorticoid treatment. This supports the notion that the patient's TBA may have been caused by the Sjögren syndrome. The characteristic features of TBA associated with Sjögren syndrome remain unclear. Therefore, further accumulation of such cases will be needed to analyze this issue properly. The prognosis is still unclear, but a few patients may develop into lymphoma. The treatment of TBA is still under trial. Asymptomatic patients usually do not require therapy and can be followed with serial pulmonary function tests and imaging. If symptomatic, the most commonly used approach is bronchoscopic intervention with mechanical debridement. In some clinical trials, steroids may improve symptoms in patients with systemic amyloidosis.^[2]^

In conclusion, although TBA is a rare complication of Sjögren syndrome it should be excluded in such patients with persistent respiratory symptoms. Computed tomography or bronchoscopy should be performed in the patients with this suspected diagnosis.

## Author contributions

**Data curation:** Jiangyun Luo.

**Writing – original draft:** Jiangyun Luo.

**Writing – review & editing:** Yongpeng Ge.
